# Probing the Conductive and Tribological Behaviors of Solid Additives in Multiply Alkylated Cyclopentanes for Sliding Electrical Contact

**DOI:** 10.3390/nano12152707

**Published:** 2022-08-06

**Authors:** Zhengfeng Cao, Qiuyu Shi, Xiangyu Ge, Shuliang Liu, Bo Wei, Ting Wang

**Affiliations:** 1 School of Advanced Manufacturing Engineering, Chongqing University of Posts and Telecommunications, Chongqing 400065, China; 2 State Grid Smart Grid Research Institute Co., Ltd., Beijing 102209, China; 3 School of Mechanical Engineering, Beijing Institute of Technology, Beijing 100081, China

**Keywords:** electrical contact resistance, graphene, carbon nanotube, conductive, tribology

## Abstract

Sliding electrical contacts need to be lubricated by conductive lubricants to perform low energy dissipation, high reliability, and long service life. This work studied the thermal stability, anti-corrosion capacity, and conductive, and tribological behaviors of several solid additives in multiply alkylated cyclopentanes (MACs), including carbon nanotubes (CNTs), multilayer graphene (MG), and silver microparticles. The results showed that all the additives possessed favorable thermal stability and corrosion resistance; in particular, CNTs and MG exhibited lower and more stable electrical contact resistance (ECR) and better lubricity abilities than Ag microparticles. Moreover, based on the characterization of the worn surfaces and the film thickness calculation, the favorable conductive and tribological properties of CNTs and MG were related to the high conductivity and specific structure of the additives and the good chemical inertness of MACs.

## 1. Introduction

A sliding electrical connector is the key component in electromechanical equipment such as electrical switches, high-speed railways, and power transmission systems, and the main function is to connect, break, or transmit electrical energy or signals [1,2,3]. Given the increasing number of sliding electrical connectors during the process of electrification engineering, the requirement for reliability and service life is also rising [4]. In the past few decades, attention has been focused on modifying materials to improve the tribological properties and reduce electrical contact resistance (ECR) [5,6,7]. Even though a series of metal-based self-lubricating materials have been explored as contact materials, these problems still exist [5,6,7]. Recent studies show that it is possible to solve these problems by the usage of lubricants [8,9,10].

Focusing attention on the lubricants that are employed to lubricate sliding electrical connectors, they not only need to have excellent electrical conductivity to reduce the transmission loss of energy or signal, but also need to have excellent tribological properties to reduce the wear of sliding electrical connectors and prolong their service life [8,9,10]. Lubricants are complex mixtures consisting of a base oil/grease and highly specialized chemicals, namely additives. The base oil/grease contributes the fundamental properties, while the additive imparts additional characteristics to the final products. Since base oil/grease usually has poor conductivity, conductive additives should be utilized to impart good conductivity to lubricants. Noble metals such as gold, silver, and palladium may be good candidates to enhance the conductive and tribological properties. They could deposit on the contact surfaces to generate a film, thereby enhancing the conductive and tribological behaviors of the sliding electrical contacts [10,11,12]. However, due to the high cost, the noble metals are not suitable for massive applications [8,9].

Owing to the rapid development of nanotechnology, carbon nanotubes (CNTs) and graphene were found one after another and they have attracted intensive attention across scientific and industrial fields. Graphene is a monolayer of carbon’s hexagon allotrope π stacked together, and CNTs are thought of as rolled sheets of graphene [13,14,15,16]. They all have excellent conductivity, novel thermal stability, outstanding tribological properties, and so forth [15,17,18]. Meanwhile, due to their large theoretical specific surface area (SSA), they may generate many contact points in lubricants, thereby improving conductivity [8,9]. Therefore, CNTs and graphene hold great promise as conductive additives in lubricants to lubricate electrical contacts.

From the previous research, the ECR is measured under a relatively low current and then is simply used as an indicator to assess the boundary lubrication film [19,20,21]. There is no excessive attention paid to the magnitudes of ECR during the friction process. On the electrical performances of connectors subjected to friction, the wear variation is not the most significant aspect but the ECR [22]. In addition, some research has also indicated that the stability of the conductive and tribological behaviors seriously affects the performance of a sliding electrical contact [5,23]. At present, much of the literature has reported that CNTs and graphene all have excellent electrical conductivity and tribological properties. However, there are few reports regarding the usage of CNTs and graphene as additives to prepare conductive lubricants, and investigating their conductive and tribological behaviors and related mechanisms under current-carrying friction conditions.

In this work, multiply alkylated cyclopentanes (MACs) are used as the base oil due to their impressive properties including chemical inertness, thermal stability, tribological properties, and so forth [24,25,26]. The ECR and tribological properties of CNTs, multilayer graphene (MG), and Ag in MACs are investigated under different loads and currents. The thermal stability and corrosion resistance of lubricants are also characterized. Meanwhile, the stability of the conductive and tribological behaviors are evaluated by the standard deviation (SD). After the friction test, the conductive and lubrication mechanisms are analyzed and discussed.

## 2. Experiment

### 2.1. Materials

MACs and multilayer graphene (MG, SSA: 647 m^2^/g) was synthesized by the State Key Laboratory of Solid Lubrication, Lanzhou Institute of Chemical Physics, Chinese Academy of Sciences (Lanzhou, China). Table 1 lists the typical properties of MACs. CNTs (outer diameter: ~50 nm, SSA: 173 m^2^/g) and Ag microparticles (diameter: ~1 μm, SSA: 6 m^2^/g) were purchased from Chengdu Organic Chemicals (Chengdu, China). In this study, 0.2 wt% additives were uniformly dispersed in MACs by sonication for 30 min.

### 2.2. Characterization of the Materials

A Q500 thermogravimetric analyzer (TGA, TA Instruments, New Castle, DE, USA) was used to characterize the thermal stability of lubricants. The heating rate was 10 °C/min and the atmosphere condition was air. The procedure of the corrosion test was as follows: At first, a piece of polished copper was immersed in lubricant and heated at 150 °C for 24 h. After that, the copper block was cleaned with ethanol, and then the corrosion resistance was compared with the polished copper block.

### 2.3. Tribological Tests

**Figure 1:**
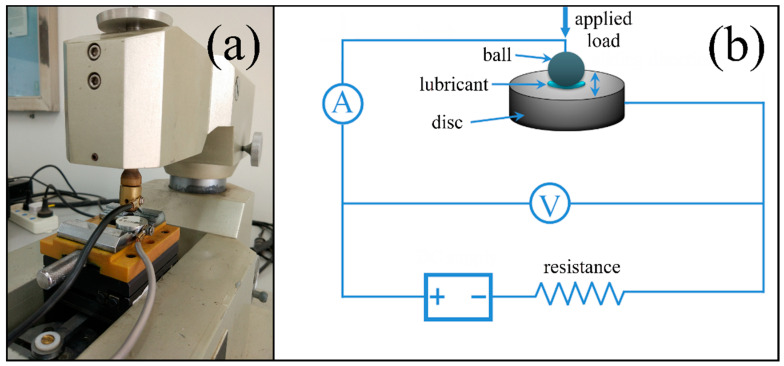
Picture (**a**) and schematic diagram (**b**) of the friction apparatus.

The current-carrying tribological tests were achieved on a friction apparatus (MFT-R4000). Figure 1 gives the picture and schematic diagram of the friction apparatus. The current and voltage of the friction interfaces was monitored and recorded in live time during the friction process. The upper ball (diameter, 5 mm) slid against the lower disc (20 mm × 20 mm × 5 mm) with a frequency of 2 Hz and an amplitude of 5 mm. The ball and disc were all made of copper (CuZn_40_, purity, >99.99%, hardness, 100–120 Hv). The load and voltage was set as three combinations including 5 N–500 mV, 5 N–1500 mV, and 20 N–1500 mV. All the discs and balls were polished with diamond paste to acquire a surface roughness of about 0.05 μm. The relative humidity and temperature was 30% and 25 °C, respectively. Before each tribological test, the balls and discs were ultrasonically washed with acetone for 10 min and then the friction region was filled with about 0.5 g lubricant. The curves of the coefficient of friction (COF) were recorded and every tribological test was repeated three times to ensure the accuracy of the experimental data. After the friction test, the electrical contact resistance (ECR) and the standard deviation (*SD*) of COFs and ECR were calculated, respectively.

### 2.4. Surface Analysis

All the discs were ultrasonically washed with acetone for 10 min. Then, a high-power optical microscope was used to measure the wear width. The morphology and element composition of the worn surface were obtained using a scanning electron microscope (SEM, EVO-18, Zeiss, Oberkochen, Germany) and an energy dispersive X-ray analyzer (EDS, Bruker, Karlsruhe, Germany). A PHI-5702 multifunctional X-ray photoelectron spectrometer (XPS, Physical Electronics, inc., Washington, D.C., WA, USA) was used to probe the elements’ chemical states on the worn surface. The binding energy of O1s is 531.0 eV, which was used as the reference.

## 3. Results and Discussion

### 3.1. Results

**Figure 2:**
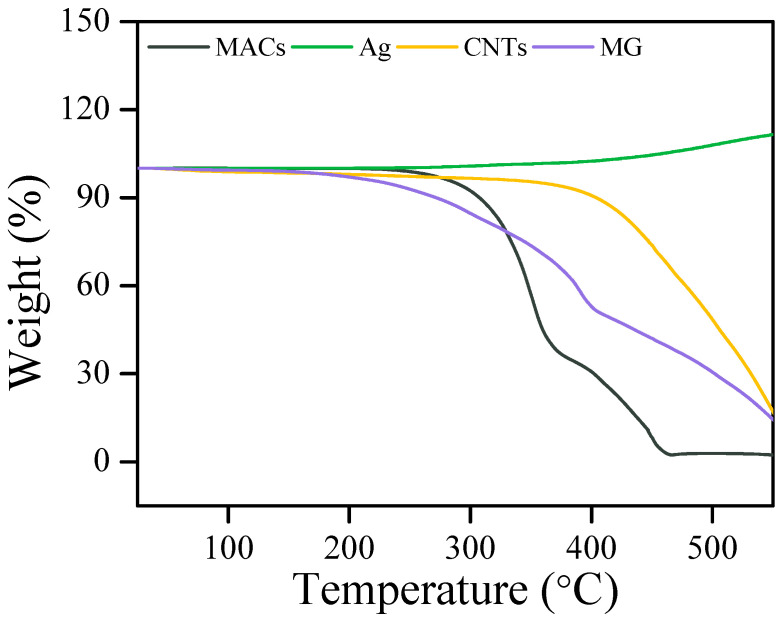
TGA curves of MACs and additives.

The TGA curves of MACs and additives are shown in Figure 2. MACs have a decomposition temperature of approximately 285 °C. Ag has a slowly increasing mass from about 300 °C, indicating that Ag is oxidized under a relatively high temperature. CNTs and MG start to decompose at about 220 °C, which is attributed to the pyrolysis of the carbon backbone and the removal of labile oxygen-containing functional groups [27,28]. In some severe cases, electrical contacts can have a temperature as high as 150 °C [29,30]. Herein, all the samples exhibited good thermal stability to meet the requirement because they have a decomposition temperature higher than 200 °C.

**Figure 3:**
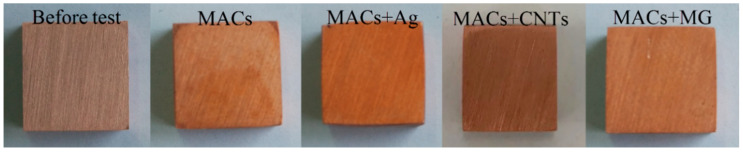
Copper blocks (20 × 20 × 5 mm) after corrosion test.

The pictures of the copper blocks after the corrosion test are shown in Figure 3. In general, compared with the polished copper, if the color of copper changed obviously, it indicated that this lubricant had a certain corrosion performance. Here, compared with the copper block before the corrosion test, the copper blocks immersed in a different lubricants all had no obvious corrosion phenomenon, indicating that the lubricants had good corrosion resistance ability.

**Figure 4:**
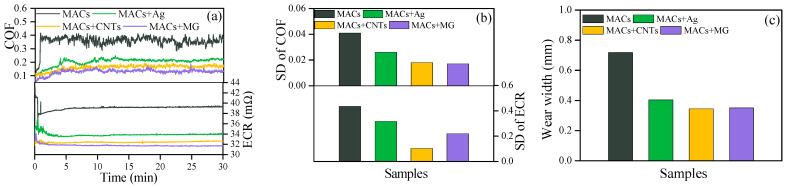
(**a**) COF and ECR, (**b**) the corresponding SD, and (**c**) wear widths on copper discs under 5 N and 500 mV at 2 Hz.

Figure 4a,b give the COF and ECR curves and the SD. It can be seen that all the additives in MACs not only significantly lower the COF and ECR, but also make them more stable. The lowest COF and ECR values are obtained by MACs + MG, reducing the COF and ECR by approximately 64% and 20% compared with MACs, respectively. Figure 4c gives the wear widths of the wear scars under the lubrication of different lubricants. Compared with pure MACs, MACs containing CNTs or MG all could reduce the wear widths by about 52% compared with MACs, indicating a superior anti-wear property.

**Figure 5:**
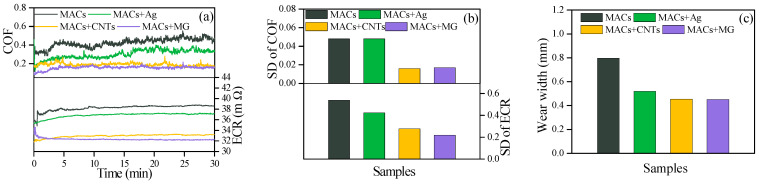
(**a**) COF and ECR, (**b**) the corresponding SD, and (**c**) wear widths on copper discs under 5 N and 1500 mV at 2 Hz.

Figure 5 presents the COF, ECR, SD, and wear widths under 5 N and 1500 mV at 2 Hz. As can be seen from Figure 5a,b, though the voltage is increased by ten times up to 1500 mV, CNTs and MG still exhibited an outstandingly low and stable COF and ECR throughout the whole friction process. The addition of Ag in MACs also made the COF and ECR lower. However, the SD values were relatively large. The wear widths of the wear scars depicted in Figure 5c suggest that the wear resistance ability of MACs was also greatly improved by adding Ag, CNTs, or MG.

**Figure 6:**
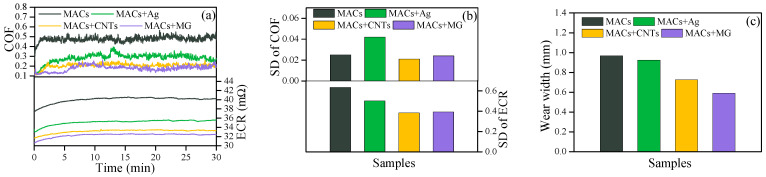
(**a**) COF and ECR, (**b**) the corresponding SD, and (**c**) wear widths on the discs under 20 N and 1500 mV at 2 Hz.

Figure 6 gives the experimental results under 20 N and 1500 mV at 2 Hz. As can be seen from Figure 6a, MACs containing additives all reduced the COF by more than 40% compared with MACs. However, Figure 6b shows that Ag exhibited a higher SD value of COF than others, indicating the friction was not stable. CNTs and MG in MACs exhibited low and stable ECR, indicating good conductive and friction reduction abilities. Figure 6c depicts that MACs containing CNTs or MG had lower wear widths than pure MACs and MACs + Ag, demonstrating a superior wear-resistant property.

### 3.2. Discussion

**Figure 7:**
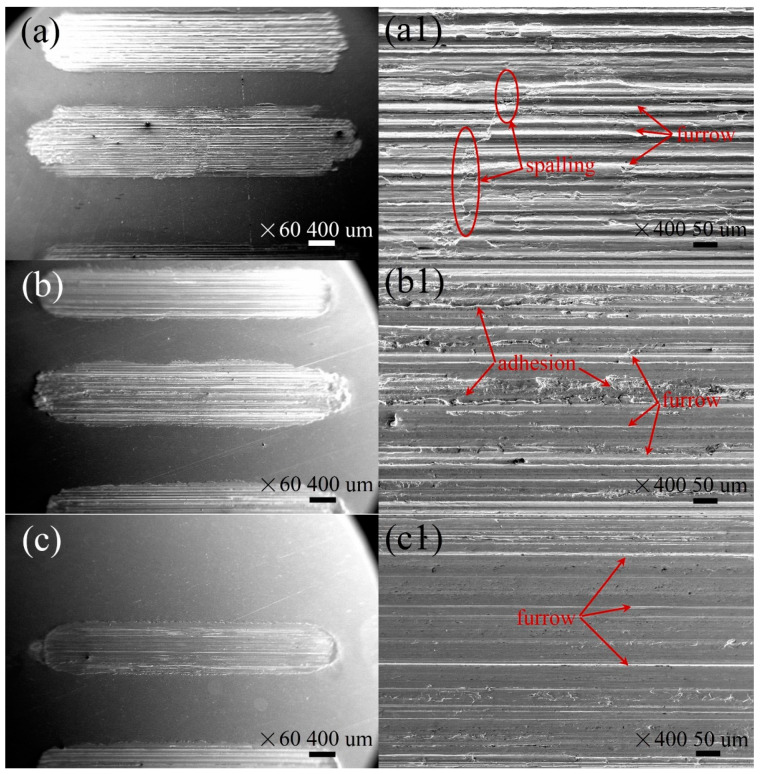

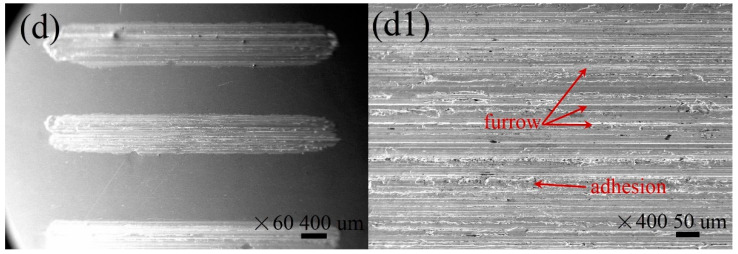
Morphologies of the worn surfaces under lubrication of (**a**,**a1**) MACs, (**b**,**b1**) MACs + Ag, (**c**,**c1**) MACs + CNTs, (**d**,**d1**) MACs + MG under 20 N and 1500 mV at 2 Hz.

To study the conductive and tribological mechanisms of these additives, several surface characterizations were employed. The morphologies of the worn surfaces under the lubrication of different lubricants were obtained by an SEM (Figure 7). The worn surface (Figure 7a,a1) lubricated by pure MACs was wider and rougher. There were a lot of deep furrows and spalling dominated by abrasive and fatigue wear on the worn surface, indicating severe wear was taking place under this condition. Figure 7b shows that Ag did not significantly reduce the wear widths. Figure 7b1 suggests that the worn surface lubricated by MACs + Ag had some shallow grooves and adhesion regions, which may be due to the abrasive wear and Ag particles depositing on the worn surface. Figure 7c,c1,d,d1 shows that the addition of CNTs or MG in MACs can reduce the wear widths and make the worn surfaces smoother, as compared with pure MACs, exhibiting an outstanding wear-resistance ability.

**Figure 8:**
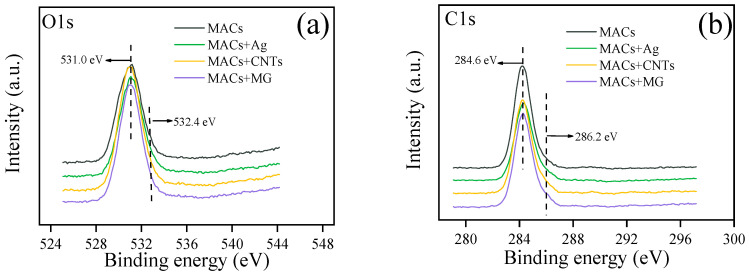

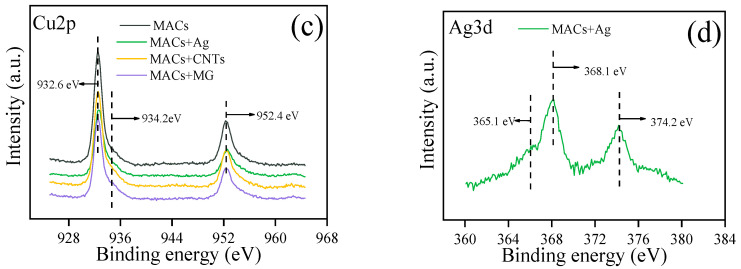
XPS of the typical elements on the worn surfaces under lubrication of MACs in the absence and presence of additives under 20 N and 1500 mV at 2 Hz. (**a**) the XPS curve of O1s, (**b**) the XPS curve of C1s, (**c**) the XPS curve of Cu2p, (**d**) the XPS curve of Ag3d.

**Figure 9:**
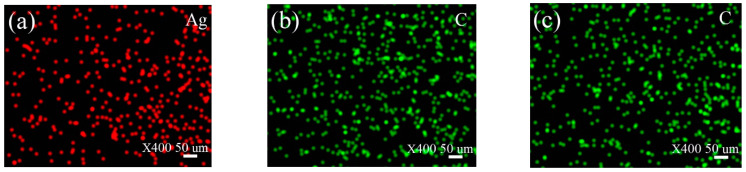
EDS elemental surface distribution of the copper worn surfaces under lubrication of (**a**) MACs + Ag, (**b**) MACs + CNTs and (**c**) MACs + MG.

The chemical state of elements on the worn surface is crucial to understanding the conductive and lubricating mechanisms; therefore, XPS is employed and Figure 8 gives the XPS spectra of O1s, C1s, Cu2p, and Ag3d. The XPS of O1s (Figure 8a) has two peaks at 531.0 eV and 532.4 eV, which belong to carbon oxides and copper oxides [31,32]. The C1s peaks at 284.6 eV and 286.2 eV shown in Figure 8b belongs to –C-C, –C-H and –C-O bonds, which are identified as derived compounds the from the lubricants [31]. Figure 8c depicts that the XPS spectra of Cu2p has peaks at 932.6 eV, 952.4 eV and 934.2 eV. Combining the XPS peaks of O1s, it can be inferred that there are metallic copper and copper oxides on the friction surface [32,33,34]. Figure 8d depicts the XPS spectra of Ag3d having sharp peaks at 368.1, 365.1 eV and 374.2 eV, which are assigned to Ag [20,35]. Combining the TGA result that Ag is oxidized under a high temperature of about 300 °C, it can be confirmed that metal Ag is deposited on the copper worn surface to enhance the conductive and lubrication performances. Figure 9 gives the EDS surface distribution images of Ag and C elements on the copper worn surfaces. It can be seen that Ag or C elements achieved high-density coverage on the copper worn surface. Under the action of pressure, solid additives can fill in the valley of surfaces and deposit on the friction surface to increase the contact area, which can reduce friction and wear. EDS characterization of the worn surfaces further provides direct evidence for the generation of a deposited lubricating film on the worn surface. XPS and EDS analysis show that a protective film is formed on the copper worn surface, which enhance the conductive and tribological behaviors throughout the friction test.

Under different loads and applied voltages, the MACs containing additives greatly make the COF, ECR, and wear width lower and more stable, as compared with pure MACs. Meanwhile, it can be found that different additives exhibit distinguished conductive and tribological behaviors. Many researches have proposed the mechanisms of solid particles as additives including the rolling effect, mending effect, polishing effect, protective film, and the others [31,36,37]. Herein, combined with the experimental results and analysis, the conductive and lubrication mechanisms for MACs containing Ag, CNTs, or MG are discussed in detail.

The similar mechanisms of Ag, CNTs, and MG in MACs can be proposed in the following two aspects: On one hand, the XPS analysis suggests that the chemical reaction protective film composed of carbon oxides, copper oxides, and so on, are all generated on the friction surfaces to improve the tribological properties. On the other hand, based on the EDS analysis and the model proposed by De-Xing Peng [38], solid additives could achieve the mending effect by filling the interspace of contact surfaces and depositing to act as a physical film. This physical film could increase the real contact area, bear the load from the ball, and disperse stress concentration, thereby improving the conductive and tribological properties.

In addition to these two aspects, a large number of experimental analyses suggest that spherical or rod-shaped nanoparticles may perform the rolling effect under boundary lubrication [38,39,40]. However, the rolling effect is dependent on the size of the additive and the thickness of the lubrication film [40]. Here, the film thickness *h* can be approximately calculated according to the H-D Equation (1) [41].

(1) $$h=2.69\frac{G^{0.53}RU^{0.67}}{W^{0.067}}\left(1-0.61e^{-0.73k}\right)$$

(2) $$\frac{1}{E^{\prime }}=\frac{1}{2}\left(\frac{1-v_{ball}^{2}}{E_{ball}}+\frac{1-v_{disc}^{2}}{E_{disc}}\right)$$

where *G* = *αE*′, *U* = *nu/E*′*R*, and *W* = *w/E*′*R*^2^. Here, *E*′ is the equivalent elastic modulus of the two friction materials, *α* is the pressure-viscosity coefficient (PVC) (11.6 GPa^−1^), *n* refers to the dynamic viscosity (DV) (0.0948 Pa·s), *w* is the applied load (5 N), *u* is the sliding speed (0.02 m/s), *k* = 1 is a coefficient, *R* is the effective radius, and *v* and *E* are the Poisson’s ratio and elastic modulus [42].

The contact area could be considered as the Hertz elastic deformation region, thus the relationship between wear scar width and the *R* can be described by Equation (3).

(3) $$\frac{d}{2}={\left(\frac{3Rw}{4E^{\prime \prime }}\right)}^{1/3}$$

Thus, *R* can be calculated by Equation (4) [39].

(4) $$R=\frac{E^{\prime \prime }d^{3}}{6w}\;$$

where *E*″ is the effective elastic modulus of the two friction materials, which could be obtained by Equation (5).

(5) $$\frac{1}{E^{\prime \prime }}=\frac{1-v_{ball}^{2}}{E_{ball}}+\frac{1-v_{disc}^{2}}{E_{disc}}$$

During the friction process under 5 N and 500 mV, the temperature of the friction fluctuated between 28 and 45 °C, which was measured using an infrared thermometer. In this study, the PVC and DV values of MACs at 30 °C are about 11.6 GPa^−1^ and 0.0948 Pa·s, respectively. After using the appropriate values of material properties and physical constants, the lubrication film thickness under 5 N at about 30 °C was determined to be approximately 34.7 nm, which could be used as a benchmark to illustrate the possible lubrication mechanisms.

The Ag and CNTs used in this study had a diameter of about 1 μm and 50 nm, respectively. As reported by Rapoport [43], spherical nanoparticles are more likely to perform a rolling effect when the size of the particle is close to the thickness of the lubrication film. Apparently, under the load of 5 N and the temperature of 30 °C, the rolling effect is not the dominant lubrication mechanism for Ag and CNTs. As known, increasing temperature leads to a reduction in PVC and DV values. According to Equation (3), reduced PVC and DV values cause a decrease in film thickness. Meanwhile, as reported by Xie [40], the lubrication film thickness may decrease with the increasing load. Therefore, under the other tested condition, where the temperature is higher than 30 °C and the load is higher than 5 N, the lubrication film thickness was lower than 34.7 nm. Thus, Ag and CNTs are more difficult to perform the rolling effect in the friction process. The above analysis suggests that the dominant lubrication mechanism for Ag and CNTs mainly depends on the “mending effect”, as mentioned above.

Our previous research suggests that when the mass fraction of solid additives are the same, the additives with a smaller size and higher specific surface area (SSA) could generate more conductive paths and form a denser protective film, thereby contributing to a better conductive and tribological performances [8,9]. CNTs (50 nm, 173 m^2^/g) have a smaller size and higher SSA than Ag (1 μm, 6 m^2^/g); therefore, CNTs could exhibit lower ECR and better tribological properties than Ag under all the tested conditions. Ag has a diameter of 1 μm which is much larger than the film thickness. When performing the mending effect, some Ag particles may also act as abrasive particles during the friction process. The worn surface lubricated by MACs + Ag shown in Figure 7b1 has obvious shallow grooves generated by abrasive wear, which may be consistent with this view. Therefore, Ag exhibits a higher and more fluctuant ECR and COF than CNTs.

In terms of MG, it is well known that platelet-shaped particles are less likely to roll between the contact interfaces, as compared with spherical or rod-shaped particles; thus, the lubricity mechanisms of MG are related to its special structure [40]. Because MG has a two-dimensional layered structure and the layers are linked by weak van der Waals bonds, it could act as a “third body” to decrease the direct contact between friction pairs and form an easily shearing lubricating film on the friction surfaces, thereby greatly enhancing the tribological properties [17,18]. MG exhibits a lower ECR than CNTs because MG has a larger specific surface area (647 m^2^/g). As depicted in Figure 9c, MG dispersed in MACs could generate more contact points to form conductive paths, resulting in a better conductive performance during the friction process.

## 4. Conclusions

A series of lubricants were prepared by blending MACs with conductive additives including Ag, CNTs, or MG. TGA and corrosion tests show that they all have good thermal stability and anti-corrosion properties. Current-carrying friction tests show that CNTs and MG greatly reduce and stabilize the COF, ECR, and wear width, as compared with Ag. Based on SEM, EDS, XPS analysis, and the lubrication film thickness calculation, the preferable conductive and tribological performances mainly depended on the synergistic effect such as the mending effect, the protective film, and so on. Given the good conductive and tribological behaviors, MACs containing CNTs or MG may hold great promise as lubricants for the sliding electrical contact.

## Figures and Tables

**Table 1:** Typical properties of MACs.

Item	Kinematic Viscosity (mm^2^/s)		Viscosity Index	Pressure-Viscosity Coefficient (GPa^−1^, 29 °C)	Dynamic Viscosity (Pa·s, 30 °C)
	40 °C	100 °C			
MACs	112	14.7	135	11.6	0.0948

## Data Availability

Not applicable.
